# Stability of refractive outcomes after hyperopic LASIK with and without Mitomycin C application: a randomized controlled trial

**DOI:** 10.1038/s41598-024-83757-x

**Published:** 2025-01-06

**Authors:** Amr Saad, Johannes Steinberg, Andreas Frings

**Affiliations:** 1 Department of Ophthalmology, Medical Faculty, University Hospital Düsseldorf, Heinrich Heine University, Moorenstraße 5, 40225 Dusseldorf, Germany; 2 Department of Ophthalmology, Stadtspital Zurich, Zurich, Switzerland; 3 Spross Research Institute, Zurich, Switzerland; 4 Department of Ophthalmology, University Medical Center, Hamburg, Germany; 5 Zentrum Sehstärke, Hamburg, Germany; 6 Augenheilkunde & Augenlaser Zentrum PD Dr. med. A. Frings, Nürnberg, Germany

**Keywords:** Refractive surgery, Laser-in-Situ-Keratomileusis, MMC, Mitomycin C, Refractive outcome, Outcomes research, Risk factors

## Abstract

To assess the efficacy, safety, and stability of refractive outcomes in hyperopic Laser-Assisted in Situ Keratomileusis (LASIK) with and without the application of Mitomycin C (MMC). This randomized, parallel group, controlled multicenter trial included 140 hyperopic eyes. The participants were randomly assigned to two groups: one receiving LASIK with mitomycin C (MMC) (*n* = 70) and the other receiving LASIK without MMC (*n* = 70). The primary outcome measures were uncorrected distance visual acuity (UDVA), corrected distance visual acuity (CDVA), and safety parameters at six months postoperatively. The statistical analysis employed t-tests, Mann-Whitney tests, and Fisher’s Exact Test, with a significance level of *p* < 0.05. All 140 eyes (70 per group) were analyzed. No statistically significant differences were identified between the two groups in postoperative UDVA, CDVA, or safety parameters (*p* > 0.05). Both groups demonstrated highly effective and safe refractive outcomes. No intraoperative complications or postoperative adverse events were observed. Age and preoperative spherical equivalent did not significantly affect outcomes. Hyperopic LASIK with and without MMC showed comparable efficacy, safety, and stability of refractive outcomes at the six-month postoperative interval. Although MMC use in hyperopic LASIK appears to be a safe procedure, it was not found to be significantly superior to conventional LASIK. Further investigation with longer follow-up periods and larger cohorts is necessary to confirm these results.

## Introduction

Over the past decades, refractive surgery has undergone significant advancements in terms of safety and efficacy resulting in increased popularity among patients^1^. However, there are still some challenges associated with the correction of refractive errors, including hyperopia, which is less predictable than myopia treatment and has a higher incidence of regression in the postoperative period^2,3^. The regression of hyperopic correction following refractive surgery is a multifactorial process that includes epithelial and stromal remodeling in the postoperative period, which mainly caused by the steeper ablation profiles and smaller ablation zones^4,5^. This has been shown to lead to increased release of cytokines, which can catalyze an inflammatory reaction^3^.

Mitomycin C (MMC) is an antimetabolite agent, which inhibits fibroblast proliferation^6^ and has been used for many years in various refractive procedures^7^. However, its use in refractive surgery has been controversial due to potential complications, such as corneal thinning and endothelial cell loss^8,9^. Despite this, numerous studies have emphasized the effectiveness of MMC in reducing the risk of corneal haze and refractive regression^10,11^. This is particularly significant in hyperopic laser treatment and high ablation depths, where the risk of refractive regression is higher.

While MMC has been predominantly used in Photorefractive Keratectomy (PRK) surgery^12^, its use in combination with Laser-Assisted in Situ Keratomileusis (LASIK) has been rarely reported in the literature. Although only two studies have reported the efficacy of MMC in hyperopic LASIK, they demonstrated promising results in improving refractive outcomes with the use of MMC^13,14^. Therefore, this study seeks to further investigate this topic in a multicenter setting comparing the refractive outcomes of two groups of patients undergoing hyperopic LASIK, with and without MMC. We aim to provide valuable insights for refractive surgeons and explore the potential benefits of MMC in refractive surgery.

## Methods

This prospective comparative randomized multicenter study was conducted at five private practices in Germany. Our study received approval from the local ethics committee at the University of Düsseldorf (2022 − 1980) and was registered on 29/08/2022 at the German Clinical Trials Register under the DRKS-ID: DRKS00029971. All methods were performed in accordance with the relevant guidelines and regulations, including the Declaration of Helsinki. All patients gave informed consent for the use of their routinely collected data for research purposes.

The study included 140 hyperopic eyes who met the inclusion criteria for refractive surgery with LASIK. The sample size was determined based on previous similar studies^13,14^. Recruitment took place from 03.04.2023 to 11.11.2023. The study included adult patients undergoing hyperopic LASIK surgery who provided informed consent, were aged 21 years or older, and presented with hyperopia between + 1.00 and + 6.00 diopters. The exclusion criteria were defined as follows: systemic diseases with impaired wound healing, other eye diseases, past refractive surgery procedures, inability to complete the follow-up, and patient objection to data analysis. We included two groups in our analysis: Group A consisted of 70 eyes that underwent hyperopic LASIK surgery with MMC (0.02%) application for 30 s after the laser ablation, and Group B included 70 eyes that underwent conventional hyperopic LASIK surgery without MMC use. A simple unrestricted randomization was employed using the random number generator function in Microsoft Excel 2017 (Microsoft, USA), which was unrestricted. The allocation sequence was generated and concealed by the trial statistician, who was not involved in the recruitment of participants or the assignment of treatments. This method ensured a randomized distribution of participants between cohorts A and B while minimizing potential randomization bias.

All eyes were targeted for emmetropia. The treatments were performed by the same experienced refractive surgeon (A.F.) using the ZEISS MEL90 (Carl Zeiss Meditec) and AMO FS60 (Johnson & Johnson) laser platforms, following the standard protocol for hyperopic Femto-LASIK. The ablation zone for all eyes was set to 7 mm and our in-house ablation nomograms were applied.

The preoperative examination was conducted two weeks prior to surgery and after a two-week break from contact lens wear, if applicable. The postoperative examination was performed at 3 and 6 months after the surgery. Visual acuity (VA) was assessed in terms of uncorrected distance visual acuity (UDVA) and corrected distance visual acuity (CDVA) at each visit as primary outcome. Refractive error was measured using subjective refraction and corneal topography was performed to monitor changes in keratometry. In addition, any complications or adverse events were documented during the postoperative visits as secondary outcomes.

Statistical analysis was conducted using R Core Team (R Foundation for Statistical Computing, 2021) to evaluate the refractive outcomes and stability of hyperopic LASIK in the patient population. Differences in preoperative and postoperative parameters were assessed using either an independent t-test or a Mann-Whitney test, depending on whether the assumptions of a parametric test were met. Normality was assessed using the Shapiro-Wilk test, homogeneity of variances was assessed using the Levene test, and outliers were identified using the box plot method. Predictability was analyzed using the least squares method. The differences in the percentage (%) of eyes within ± 0.5 D between the two groups were analyzed using Fisher’s Exact Test. The level of statistical significance was set at *p* < 0.05.

## Results

In this prospective multicentral study, we evaluated the refractive outcomes of 140 eyes treated with hyperopic Femto-LASIK (FS-LASIK). The group was composed of 40% men and 60% women, with a mean age of 37 years (95%SD ± 10 years) ranging from 20 to 54 years. No intraoperative complications or postoperative adverse events were seen in either of the groups.

Table 1 summarizes the preoperative descriptive data of the two treatment groups and the total cohort in terms of sphere, cylinder, and spherical equivalent (SE). Preoperatively, the mean refractive error expressed in SE in the total group was 3.18 D (95%SD ± 0.54D), with a mean sphere of 2.92 D (95%SD ± 0.54D) and a mean cylinder of -0.53 D (95%SD ± 0.24D). Table 2 presents the same parameters postoperatively, as well as UDVA and CDVA. The mean SE decreased to 0.23 D (95%SD ± 0.35D) during the follow-up period.

**Table 1:** Preoperative descriptive data (subjective refraction).

Parameter	LASIK + MMC (*N* = 70)	LASIK without MMC (*N* = 70)	Total (*N* = 140)	*p*-value
Sphere (D)				W = 2411.5, *p* = 0.873, *r*=-0.014^a^
Range	2.00, 4.00	2.00, 4.00	2.00, 4.00	
Mean (SD)	2.91 (0.62)	2.93 (0.46)	2.92 (0.54)	
Median (Q1,Q3)	3.00 (2.50, 3.25)	3.00 (2.50, 3.25)	3.00 (2.50, 3.25)	
Cylinder (D)				W = 2634.5, *p* = 0.417, *r*=− 0.069^a^
Range	− 1.00, 0.00	− 1.00, -0.25	− 1.00, 0.00	
Mean (SD)	− 0.51 (0.25)	− 0.54 (0.23)	− 0.53 (0.24)	
Median (Q1,Q3)	− 0.50 (-0.75, -0.25)	− 0.50 (-0.75, -0.50)	− 0.50 (-0.75, -0.25)	
Spherical Equivalent (D)				W = 2390, *p* = 0.804, *r*=− 0.021^a^
Range	2.12, 4.25	2.38, 4.38	2.12, 4.38	
Mean (SD)	3.16 (0.61)	3.20 (0.47)	3.18 (0.54)	
Median (Q1,Q3)	3.19 (2.78, 3.62)	3.25 (2.78, 3.50)	3.25 (2.75, 3.62)	

LASIK: Laser-Assisted in Situ Keratomileusis, MMC: Mitomycin C, D: diopter, SD: standard deviation, Q1: first quartile, Q3: third quartile.

^a^Mann-Whitney test.

^b^Independent t-test.

**Table 2:** Descriptive data (subjective refraction) and visual acuity 6 months postoperatively.

Parameter	LASIK + MMC (*N* = 70)	LASIK without MMC (*N* = 70)	Total (*N* = 140)	*p*-value
Sphere (D)				W = 1788, *p* = 0.004, *r*=− 0.242^a^
Range	− 0.50, 1.00	− 0.50, 1.50	− 0.50, 1.50	
Mean (SD)	0.20 (0.31)	0.38 (0.36)	0.29 (0.35)	
Median (Q1,Q3)	0.25 (0.00, 0.25)	0.25 (0.25, 0.50)	0.25 (0.00, 0.50)	
Cylinder (D)				W = 2875.5, *p* = 0.039, *r*=− 0.174^a^
Range	− 0.75, 0.00	− 0.75, 0.00	− 0.75, 0.00	
Mean (SD)	− 0.10 (0.17)	− 0.15 (0.18)	− 0.12 (0.18)	
Median (Q1,Q3)	0.00 (− 0.25, 0.00)	0.00 (− 0.25, 0.00)	0.00 (− 0.25, 0.00)	
Spherical Equivalent (D)				W = 1885.5, *p* = 0.017, *r*=− 0.202^a^
Range	− 0.62, 1.00	− 0.50, 1.50	− 0.62, 1.50	
Mean (SD)	0.15 (0.31)	0.30 (0.37)	0.23 (0.35)	
Median (Q1,Q3)	0.25 (0.00, 0.25)	0.25 (0.12, 0.50)	0.25 (0.00, 0.38)	
CDVA				W = 2407, *p* = 0.838, *r*=− 0.017^a^
Range	− 0.10, 0.00	− 0.10, 0.00	− 0.10, 0.00	
Mean (SD)	− 0.02 (0.03)	− 0.03 (0.04)	− 0.03 (0.04)	
Median (Q1,Q3)	0.00 (− 0.04, 0.00)	0.00 (− 0.10, 0.00)	0.00 (− 0.04, − 0.00)	
UDVA				W = 2402.5, *p* = 0.730, *r*=− 0.029^a^
Range	0.00, 0.05	− 0.10, 0.10	− 0.10, 0.10	
Mean (SD)	0.00 (0.01)	0.00 (0.03)	0.00 (0.02)	
Median (Q1,Q3)	0.00 (0.00, 0.00)	0.00 (0.00, 0.00)	0.00 (0.00, 0.00)	

CDVA: corrected distance visual acuity, UDVA: uncorrected distance visual acuity, LASIK: Laser-Assisted in Situ Keratomileusis, MMC: Mitomycin C, D: diopter, SD: standard deviation, Q1: first quartile, Q3: third quartile.

^a^Mann-Whitney test.

There were no significant differences in any of the parameters between the two treatment groups preoperatively (*p* > 0.05). However, postoperatively, there was a significant difference in the residual refraction error (postoperative SE) between group A and B (*p* = 0.017). The LASIK with MMC eyes (Group A) were more accurately corrected compared to the LASIK without MMC eyes (Group B), which were slightly under-corrected (Q1, Q3: 0.00, 0.25 vs. 0.12, 0.50; Table 2). However, there was no significant difference in CDVA and UDVA between the two groups in the postoperative follow-up visits.

**Fig. 1:**
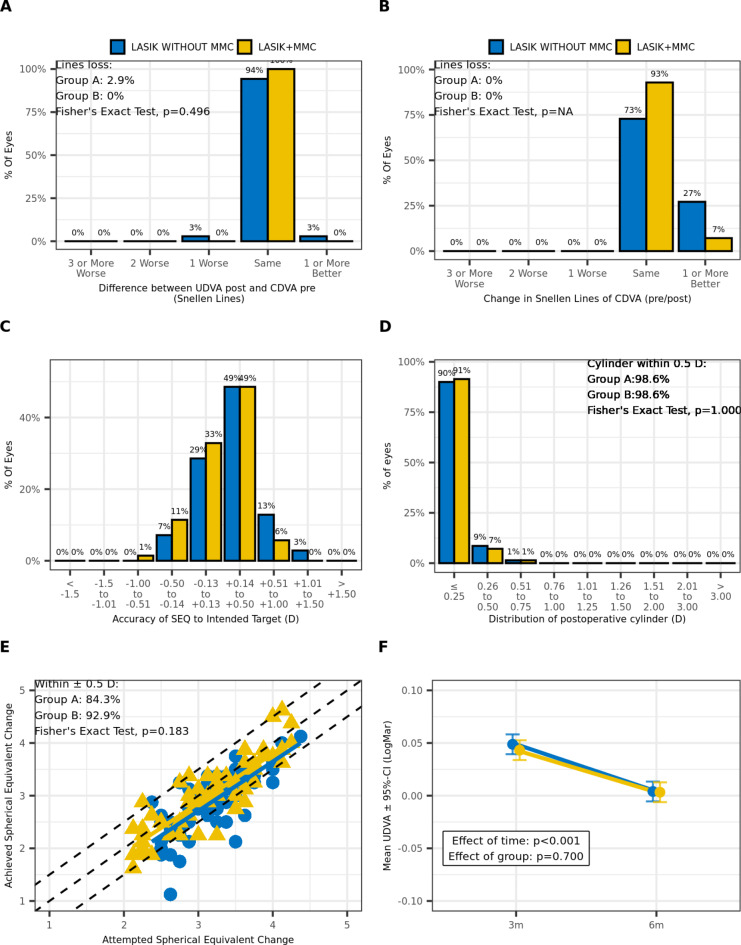
Standard graphs reporting visual outcomes for treatment groups (Group A: LASIK with MMC; Group B: LASIK without MMC): (**A**) Efficacy graph, (**B**) Safety graph, (**C**) Accuracy graph, (**D**) Residual cylinder graph; (**E**) Predictability graph; LASIK: Laser-Assisted in Situ Keratomileusis, MMC: Mitomycin C, UDVA: uncorrected distance visual acuity, CDVA: corrected distance visual acuity, SEQ: spherical equivalent (F) Stability graph; 3 m: 3 months postoperatively, 6 m: 6 months postoperatively.

The refractive outcomes were reported using five Standard Graphs (Fig. 1) as proposed by Reinstein et al.^15^. The Efficacy graph (Fig. 1A) shows postoperative UDVA in relation to preoperative CDVA, whereas the safety graph (Fig. 1B) illustrates the postoperative CDVA in relation to preoperative CDVA. The results indicate highly effective and safe refractive outcomes for both surgical procedures, with no significant differences between the two groups. Only two eyes (3%) in Group B experienced a loss of one Snellen line in terms of postoperative UDVA, which was not clinically relevant. In general, all eyes in both groups had the same CDVA or better, postoperatively. The accuracy plot diagram (Fig. 1C) demonstrates that 93% of eyes in Group A and 85% of eyes in Group B were within ± 0.5D SE postoperatively. Figure 1D indicates that most eyes had a residual cylinder of 0.5D or less after LASIK treatment (98.6% in each group). Additionally, the predictability graph (Fig. 1E) revealed no significant differences between the treatment groups regarding the attempted SE in relation to the achieved SE, as confirmed by the Fishers Exact Test (*p* = 0.18). However, Group B exhibited a slightly lower proportion of eyes achieving ± 0.5D of SE postoperatively (84.3% vs. 92.9%). Finally, the stability analysis of refractive outcomes showed a significant improvement (*p* < 0.001) over the 6-month follow-up period, indicating promising long-term results (Fig. 1F).

**Fig. 2:**
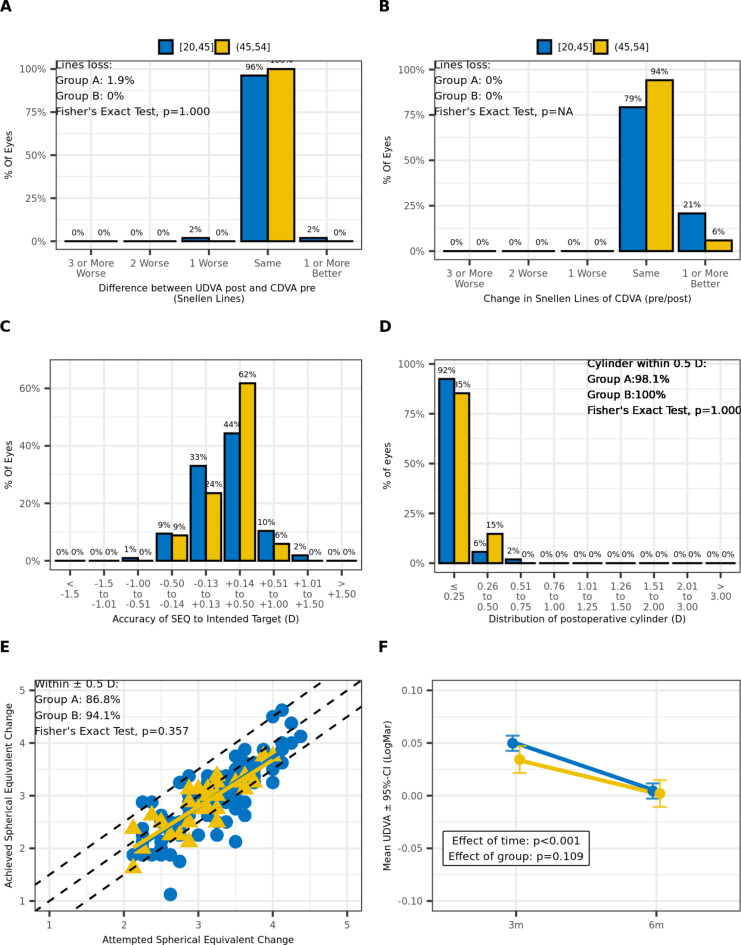
Standard graphs reporting visual outcomes for age-related groups (Group A: 20–45 years old, Group B: 45–54 years old): (**A**) Efficacy graph, (**B**) Safety graph, (**C**) Accuracy graph, (**D**) Residual cylinder graph; (**E**) Predictability graph; LASIK: Laser-Assisted in Situ Keratomileusis, MMC: Mitomycin C, UDVA: uncorrected distance visual acuity, CDVA: corrected distance visual acuity, SEQ: spherical equivalent (F) Stability graph; 3 m: 3 months postoperatively, 6 m: 6 months postoperatively.

Furthermore, we categorized the study population into two age groups: 20-45- and 45-54-years old patients. Our analysis revealed that there were no significant differences with respect to age in both treatment groups (Tables 3 and 4). Figure 2 displays the same graphs and results as the previous analysis, with eyes categorized based on age confirming the same findings.

**Table 3:** Preoperative descriptive data (subjective refraction).

Parameter	Age 20–45 y.o. (*N* = 106)	Age 45–54 y.o. (*N* = 34)	Total (*N* = 140)	*p*-value
Sphere (D)				W = 1905.5, *p* = 0.613, *r*=− 0.043^a^
Range	2.00, 4.00	2.00, 4.00	2.00, 4.00	
Mean (SD)	2.93 (0.55)	2.87 (0.51)	2.92 (0.54)	
Median (Q1,Q3)	3.00 (2.50, 3.25)	3.00 (2.50, 3.25)	3.00 (2.50, 3.25)	
Cylinder (D)				W = 1683, *p* = 0.542, *r*=− 0.052^a^
Range	− 1.00, − 0.25	− 1.00, 0.00	− 1.00, 0.00	
Mean (SD)	− 0.53 (0.23)	− 0.51 (0.29)	− 0.53 (0.24)	
Median (Q1,Q3)	− 0.50 (− 0.75, − 0.31)	− 0.50 (− 0.75, − 0.25)	− 0.50 (− 0.75, − 0.25)	
Spherical Equivalent (D)				*p* = 0.434, *r* = 0.100^b^
Range	2.12, 4.38	2.12, 4.00	2.12, 4.38	
Mean (SD)	3.20 (0.56)	3.12 (0.49)	3.18 (0.54)	
Median (Q1,Q3)	3.25 (2.75, 3.62)	3.19 (2.88, 3.44)	3.25 (2.75, 3.62)	

y.o.: years old, D: diopter, SD: standard deviation, Q1: first quartile, Q3: third quartile.

^a^Mann-Whitney test.

^b^Independent t-test.

**Table 4:** Descriptive data (subjective refraction) and visual acuity 6 months postoperatively.

Parameter	Age 20–45 y.o. (*N* = 106)	Age 45–54 y.o. (*N* = 34)	Total (*N* = 140)	*p*-value
Sphere (D)				W = 1801, *p* = 0.998, *r* = 0.000^a^
Range	− 0.50, 1.50	− 0.25, 0.75	− 0.50, 1.50	
Mean (SD)	0.29 (0.37)	0.29 (0.25)	0.29 (0.35)	
Median (Q1,Q3)	0.25 (0.00, 0.50)	0.25 (0.06, 0.50)	0.25 (0.00, 0.50)	
Cylinder (D)				W = 1790.5, *p* = 0.950, *r*=− 0.005^a^
Range	− 0.75, 0.00	− 0.50, 0.00	− 0.75, 0.00	
Mean (SD)	− 0.12 (0.17)	− 0.12 (0.19)	− 0.12 (0.18)	
Median (Q1,Q3)	0.00 (− 0.25, 0.00)	0.00 (− 0.25, 0.00)	0.00 (− 0.25, 0.00)	
Spherical Equivalent (D)				W = 1747.5, *p* = 0.790, *r*=− 0.022^a^
Range	− 0.62, 1.50	− 0.25, 0.75	− 0.62, 1.50	
Mean (SD)	0.23 (0.37)	0.22 (0.26)	0.23 (0.35)	
Median (Q1,Q3)	0.25 (0.00, 0.38)	0.25 (0.03, 0.38)	0.25 (0.00, 0.38)	
CDVA				W = 1460, *p* = 0.056, *r*=− 0.162^a^
Range	− 0.10, 0.00	− 0.10, 0.00	− 0.10, 0.00	
Mean (SD)	− 0.03 (0.04)	− 0.01 (0.03)	− 0.03 (0.04)	
Median (Q1,Q3)	0.00 (− 0.04, 0.00)	0.00 (− 0.03, 0.00)	0.00 (− 0.04, 0.00)	
UDVA				W = 1921.5, *p* = 0.308, *r*=− 0.086^a^
Range	− 0.10, 0.10	− 0.00, 0.05	− 0.10, 0.10	
Mean (SD)	0.00 (0.02)	0.00 (0.01)	0.00 (0.02)	
Median (Q1,Q3)	0.00 (0.00, 0.00)	0.00 (0.00, 0.00)	0.00 (0.00, 0.00)	

y.o.: years old, CDVA: corrected distance visual acuity, UDVA: uncorrected distance visual acuity, D: diopter, SD: standard deviation, Q1: first quartile, Q3: third quartile.

^a^Mann-Whitney test.

**Fig. 3:**
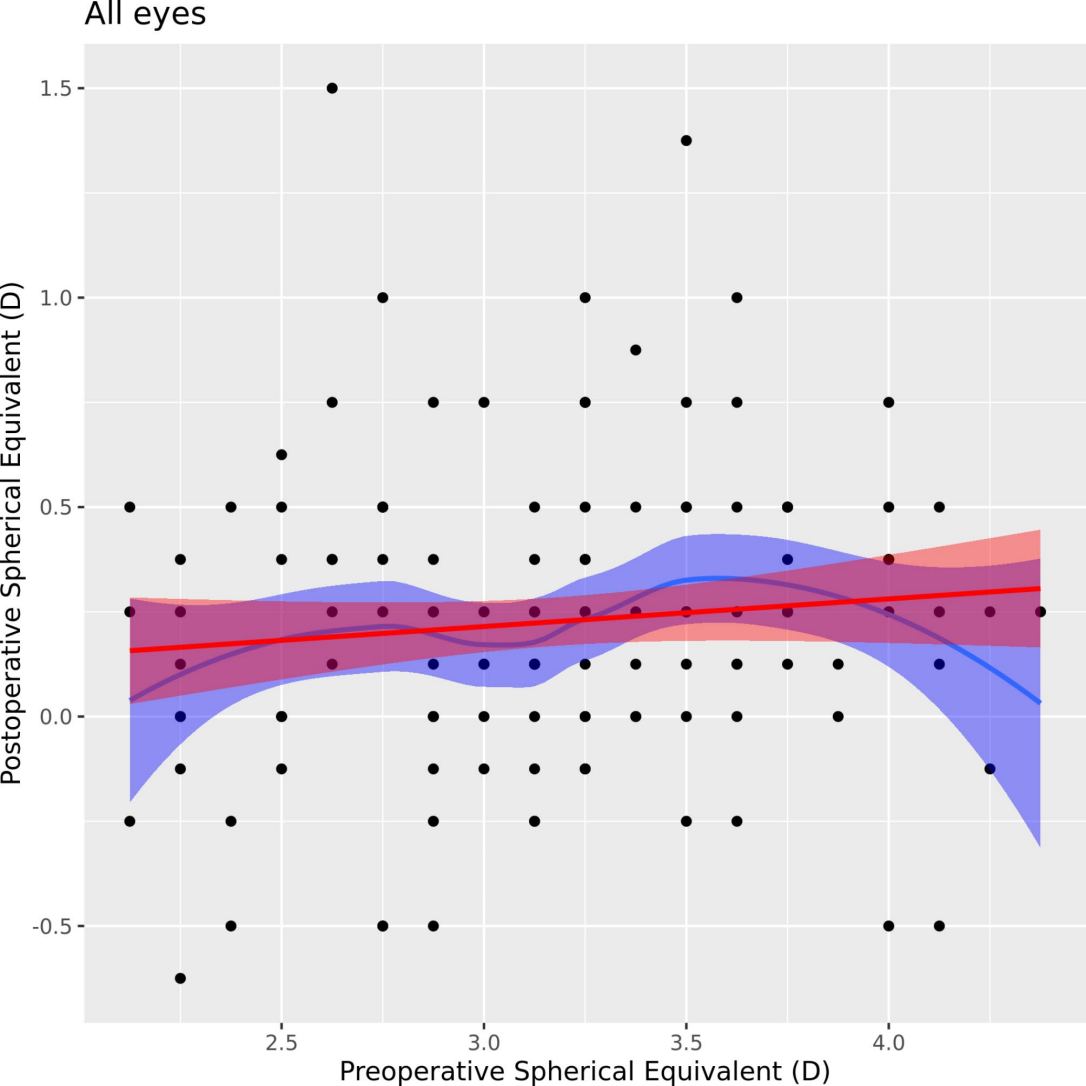
Correlation graph of pre- and postoperative spherical equivalent (SEQ).

**Fig. 4:**
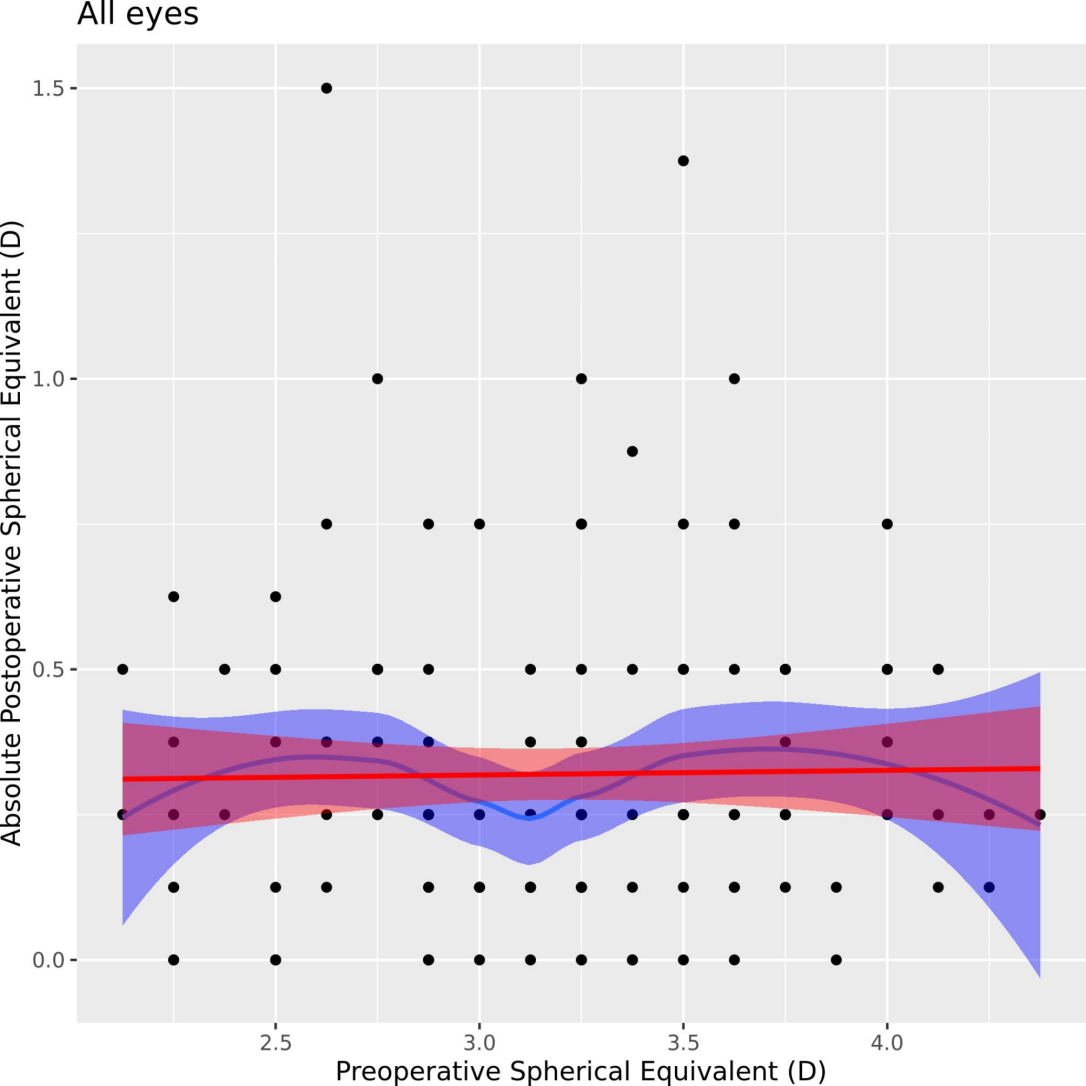
Correlation graph of preoperative and absolute postoperative SEQ.

Additionally, we observed a very low correlation between preoperative and postoperative SE expressed in a low coefficient of determination (R^2^ = 0.01, *p* = 0.226), which suggests that preoperative SE was not a significant predictor of refractive outcome (postoperative SE). These results are demonstrated by Figs. 3 and 4.

## Discussion

In the current study, we aimed to compare the safety and efficacy of using MMC in hyperopic LASIK surgery with a control group that did not receive MMC. The refractive outcomes of both groups were evaluated, and no significant differences were found in terms of efficacy, safety, residual cylinder, predictability, accuracy and stability after six months of follow-up.

It is worth noting that previous studies have shown promising results in improving refractive outcomes with the use of MMC in hyperopic LASIK surgery, even though there has been limited research on this topic^13,14^. Moawad et al. demonstrated that the MMC group had a higher efficacy in terms of uncorrected distance visual acuity (UDVA) after 12 months of follow-up, particularly in the high hyperopia group, where this effect was more significant^13^. In addition, they evaluated the topographic results groups and observed higher keratometry values and greater corneal thickness, which can be related to higher keratocyte proliferation in the hyperopic eyes treated without MMC. These findings support the well-known cell-inhibiting effect of MMC, which has been extensively discussed in the literature. However, this aspect is beyond the scope of our current investigation.

Our investigation did not find any significant difference in efficacy between the MMC and non-MMC group, which aligns with previous findings of another study that found slightly better outcomes in the MMC group after a 15-month follow-up period, although the differences were not (clinically) significant^14^. However, we did observe a significant difference in the postoperative residual refraction error (postoperative SE) between the two groups (*p* = 0.017), with the MMC group (Group A) showing more accurate correction than the LASIK without MMC group (Group B). During the postoperative follow-up visits, there was no significant difference in CDVA and UDVA between the two groups. However, the 6% higher proportion of patients achieving the intended outcome in the MMC group may be clinically relevant. This suggests that the use of MMC could potentially improve the visual outcomes for some hyperopic LASIK patients, even if the overall refractive error correction was similar between the two groups. Notably, the scatter plot (Fig. 1E) shows also a tighter clustering of refractive results around the intended correction in the LASIK eyes treated with MMC. Differences in surgical technique between our study and previous research may account for variations in outcomes. Moawad et al. performed microkeratome LASIK and applied MMC for 10 s, whereas in our study, we used FS-LASIK and applied MMC for 30 s in Group A. Furthermore, differences in the treatment nomograms, ablation zone, refraction assessment methods could lead to inaccurate comparisons between studies.

It is important to note that the use of MMC in refractive surgery has been controversial due to potential complications such as corneal thinning and endothelial cell loss^8,9^. However, previous studies have shown that MMC can effectively reduce the risk of corneal haze and regression of in PRK eyes^12^, which explains its increasing popularity in refractive surgery. Since stromal regression is due to keratocytic remodeling and not related to biomechanical stromal remodeling, regression after hyperopic LASIK should not be as prominent as after hyperopic PRK^3^. Our study found no significant differences in terms of safety between the MMC and non-MMC group, with no Snellen line loss, retreatments, or postoperative complications in the MMC group, which is consistent with prior research^10,16^.

Overall, our study provides valuable insights into the safety and efficacy of MMC in hyperopic LASIK surgery. While our study did not find significant differences in refractive outcomes between the two groups, we observed lower predictability and a slight under-correction in the Group B during the follow-up period, although not clinically significant. These results suggest that MMC could be beneficial in reducing the risk of topographically documented regression. Future research could investigate varying concentrations and exposure durations of MMC to determine if there is an unexplored dose-response relationship when using it in the setting of hyperopic PRK.

In our study, we have also analyzed patient-related factors and their potential associations with refractive outcomes, which is a unique aspect of our research. Age is known to be a potential confounding factor for refractive regression^17,18^. In our investigation, there were no age-related differences observed in the cohort. This suggests that the use of MMC in hyperopic LASIK surgery may be beneficial for various age groups. While previous studies have suggested a strong association between hyperopia magnitude and refractive regression^19^, we observed no correlation between preoperative and postoperative spherical equivalent (SE) values in our study. We therefore conclude that hyperopic LASIK in combination with MMC is a safe and effective treatment option for hyperopic eyes with a broad range of age and refractive errors, providing stable refractive outcomes at least for the first six months.

A limitation of this study is the relatively short follow-up period of up to 6 months and the small cohort. While we did not observe any significant differences in the outcomes between the two groups during this period, a longer follow-up may be necessary to fully evaluate the course of refractive stability and any potential differences between the groups, especially as hyperopic laser treatment is associated with the risk of epithelial regression and loss of refractive effect over time^20^. A further limitation of this study is the reliance on subjective manifest refraction, rather than cycloplegic refraction, to assess refractive outcomes. This could have resulted in the masking of latent hyperopia in rare cases. Future studies with longer follow-up periods and larger cohorts are needed to provide more conclusive evidence on the effectiveness of hyperopia treatment with LASIK and MMC.

In conclusion, the results of our study indicate that the use of MMC in hyperopic LASIK surgery did not lead to significant differences in refractive outcome parameters compared to the control group without MMC, suggesting that hyperopic LASIK with MMC is a safe and effective treatment option for hyperopia across different age groups and refractive errors.

## Data Availability

Data sharing is restricted by local ethical guidelines. Please contact the corresponding author (A.F.) for further information.
